# Reproducibility Assessment of Enzyme-Linked Immunosorbent Assays to Detect Anti-HPV16 L1-Specific IgG1, IgG3, IgA, and IgM Antibodies

**DOI:** 10.3390/vaccines12101108

**Published:** 2024-09-27

**Authors:** Ken Matsui, Heidi Anne Hempel, Gloriana Shelton, Rebecca Ocampo, Troy J. Kemp, Yuanji Pan, Ligia A. Pinto

**Affiliations:** 1 Vaccine, Immunity and Cancer Directorate, Frederick National Laboratory for Cancer Research, Frederick, MD 21702, USA; 2 Applied/Developmental Research Directorate, Frederick National Laboratory for Cancer Research, Frederick, MD 21702, USA; 3 Digital Transformation Solutions/Civil Health, Leidos, Bethesda, MD 20817, USA; 4 Agencia Costarricense de Investigaciones Biomédicas (ACIB-FUNIN), San José 10108, Costa Rica

**Keywords:** HPV, ELISA, isotypes

## Abstract

Background/Objectives: Enzyme-linked immunosorbent assays (ELISAs) have been used to measure anti-human-papillomavirus (HPV) immunoglobulin IgG. The goal of this study was to evaluate the reproducibility of ELISAs measuring different HPV immunoglobulin isotypes, IgG1, 2, 3, and 4, IgA, and IgM, against HPV16. Methods: Seventy-two serum samples collected from participants in the Costa Rica HPV Vaccine Trial (CVT) and immunized with bivalent HPV vaccine (2vHPV) were used for reproducibility assessment. IgG2 and IgG4 levels were too low to be detected. Levels of IgG1, IgG3, IgA, and IgM were measured, and the data were used to calculate intraclass correlation coefficients (ICCs) and coefficients of variation (CVs). Results: CVs were assessed between technicians (12.8–22.7%) and across days (6.2–30.6%). The overall CVs ranged from 7.7–31.1%. IgM ELISA showed higher CVs (15.8–31.1%) than IgG1, IgG3, and IgA (6.2–22.7%). All ICC values were >98.7%. IgG3 was detected in all samples, while IgG1 and IgA had >86.3% detectability and IgM had 62.1% detectability. Pearson correlational analyses between different antibodies all showed significant correlations (*p* ≤ 0.001), except when comparing IgGs or IgA to IgM (*p* = 0.29–0.53). Conclusions: Our data showed that these ELISAs are reproducible and detect isotype antibodies to HPV16 L1 across a range of concentrations in 2vHPV-vaccinated participants.

## 1. Introduction

Human papillomaviruses (HPVs) are the major etiological agents responsible for the development of pre-cancerous and cancerous lesions of the cervix, as well as other anogenital cancers and oropharyngeal cancers [1]. HPV16 and 18 are responsible for approximately 70% of cervical cancers, while another 20% are believed to be caused by five other HPV types (HPV31, 33, 45, 52, and 58) [2,3,4]. The bivalent HPV vaccine (2vHPV) is formulated to protect against HPV16 and 18. Meanwhile, the quadrivalent one (4vHPV) provides protection against these two major oncogenic types as well as low-risk HPV6 and 11 (which cause anogenital warts), and the nonavalent vaccine (9vHPV) targets HPV6, 11, 16, 18, 31, 33, 45, 52, and 58 [3].

Immunization with three doses of any of the three approved vaccines results in seroconversion in 93–100% of participants by 7 months after the first dose and can provide protection against new infections and associated lesions [3]. Durability of one dose of 2vHPV has been demonstrated, such that a single immunization was enough to maintain protection against infection as well as elevated levels of HPV16 and HPV18-specific total IgG for up to 11 years [5,6,7].

In 2022, the World Health Organization (WHO) changed the recommendations for HPV vaccination from a 3-dose to a 1–2-dose schedule in girls 9–14 years old [8]. These changes were made after non-inferiority of the lower dose schedule was demonstrated using immunobridging studies, where vaccine effectiveness and antibody (mainly IgG) levels were measured and compared in clinical study participants who had received 1–3 doses of the various HPV vaccines [5,8,9,10,11,12,13,14,15,16,17,18,19,20]. One of the most commonly used assays for measuring anti-HPV antibodies in these studies is the ELISA, which measures the levels of antibodies in serum samples that bind to specific target antigens. Assays used in clinical or large-scale public health decisions must undergo extensive validation procedures to demonstrate assay reliability, specificity, and sensitivity. However, how different antibody isotypes can affect these metrics is not yet extensively explored in the HPV serology.

For the purposes of this study, ELISAs were developed to detect different anti-HPV16 L1 IgG subclasses, IgA, and IgM antibodies, and assay reproducibility was assessed using sera of 2vHPV immunized individuals from the CVT study. These assays were subsequently used to further characterize the humoral immune responses to 2vHPV vaccination.

## 2. Materials and Methods

### 2.1. Serum Samples

Serum samples from non-immunized or self-reported 4vHPV-immunized donors (range: 20–61 years of age) were collected by Occupational Health Services at the National Institutes of Health (NIH) National Cancer Institute (NCI) at Fort Detrick, MD, under the Research Donor Protocol (RDP). Participants were healthy employees at the NCI-Frederick facilities that donated blood samples for in vitro laboratory research. The protocol is listed under NIH protocol number OH99CN046 and NCT number NCT00339911. These OHS samples were used as verified seronegative or seropositive samples for reagent optimization.

Sera from participants in the CVT (NCT00128661), a randomized clinical trial of a 2vHPV (HPV 16/18 L1 VLP/AS04), were used for the assessment of reproducibility of different ELISAs. In the CVT protocol [21], women aged 18–25 years randomized to the vaccine arm received 3 intramuscular (deltoid) injections of 2vHPV over ~6 months. Samples were collected at 0 months (pre-vaccination), then, at 1, 6, and 12 months post-vaccination. This study was conducted in Costa Rica by the National Cancer Institute (NCI), National Institutes of Health (NIH) in Bethesda, Maryland, USA and the Costa Rican Agency for Biomedical Research, INCIENSA Foundation, San Jose, Costa Rica. The study, including the consent procedure, was reviewed and approved by Institutional Review Boards at the NCI and in Costa Rica. The study team followed human experimental guidelines for conducting clinical research from the US Department of Health and Human Services and Costa Rican laws in accordance with principles expressed in the Declaration of Helsinki. All subjects gave written informed consent prior to participation. The original signed consent form was filed in the subject’s medical record and a copy of the signed consent form was given to the participant.

One hundred and twenty CVT samples were selected for reproducibility assessment in this study, including twenty-five pre-vaccination samples from “naive” individuals (who were seronegative for HPV16 and HPV18 antibodies and negative for HPV DNAs) and ninety-five post-vaccination seropositive samples (N = 70, post-first dose, collected 1 month post-vaccination; N = 25, post-third dose, collected 6 months post-last dose) for 2vHPV.

A pool of other CVT samples not included in the 120 CVT samples described above was used to generate a standard for each isotype. The standard was initially assessed with the following criteria: (1) the OD value of the first serially diluted standard must be >2.2, and (2) the back-fit dose of the second serially diluted standard must be in the range of 90–110% [(experimental concentration/theoretical concentration) × 100]. Each isotype standard was assigned an arbitrary concentration (ELISA unit per milliliter (EU/mL)) based on the concentration of the standard within the total IgG assay and assigned a theoretical concentration for each isotype adjusted according to the dilution factors used to generate a standard curve. The same pool of sera was also used to generate positive controls and were tested across plates to assess assay validity.

### 2.2. Optimization of Reagents

To generate a standard and positive controls for each antibody type, the standard was tested at different starting dilution factors followed by subsequent 2-fold serial dilutions. Different concentrations of horseradish peroxidase (HRP)-conjugated secondary antibodies were also tested for each dilution of serum standard. Working dilution factors of serum standard for each antibody type and secondary antibody concentrations were determined for each assay as the combination producing minimal reactivity to negative samples (HPV-seronegative OHS serum samples tested at a starting dilution of 1:100) while generating serum standard curves between ~0.02 and 3.0 optical density (OD).

IgG2 and IgG4 were not detected at appreciable levels in serum, therefore total IgG was purified from a serum standard as well as an OHS sample known to contain high levels of anti-HPV16 L1 total IgG antibodies using a Protein G column. Protein G-column purified IgGs were enriched by using HPV16 L1 VLP antigen-coated agarose beads. Both IgG2 and IgG4 were detectable in IgG eluted from the beads. IgG2 and IgG4 were not pursued further in these studies.

The resulting dilution factors of serum standard were 1:4200 for IgG1, 1:3150 for IgG3, 1:200 for IgA, and 1:300 for IgM. For total IgG assay, the standard was diluted to 1:8400. HRP-conjugated secondary antibodies were used at 150 ng/mL (IgG1), 233 ng/mL (IgG3), 67 ng/mL (IgA), 40 ng/mL (IgM), and 16.7 ng/mL (total IgG). The same pool of sera was also used to generate positive controls at dilutions of 1:3150 (IgG1), 1:4200 (IgG3), 1:1050 (IgA and IgM).

### 2.3. Setting the Cut-Off Concentration

Assay cut-off concentrations (EU/mL) were determined for each ELISA using the 25 naive samples from the CVT and was defined as 3 times the standard deviation (SD) of the geometric mean concentration of said naive samples [22]. The calculated cut-off concentrations were 12 (IgG1), 1.25 (IgG3), 0.48 (IgA), and 4.79 (IgM). Samples that were below these values were defined as non-detectable and were assigned half the cut-off value for each assay.

### 2.4. ELISAs

The production of HPV16 L1 VLP was carried out as previously described [23,24]. The DNA used to produce HPV16 L1 VLP was p16L1h [25] (NCBI accession number AJ313179). A well-established ELISA system that we have been employing to measure HPV16 L1-specific total IgG [26] was used as a template to develop new ELISAs. General criteria used in the assays were as follows: (1) The four-parameter logistic (4-PL) curve fit had to be ≥0.99 for the standard curves; (2) change in OD values of serially diluted samples, standards, and positive controls had to be ≥30%; and (3) coefficient of variation (CV) of interpolated concentrations on the standards needed to be ≤30% after adjusting for the dilution factor. The concentrations were determined by calculating the average of all interpolated dilution points that fell within the working range of the standard curves. Those with less than two interpolated points were considered non-detectable and were arbitrarily assigned half the cut-off values for each ELISA. Standard curves were generated using the 4-PL curve equation in SoftMaxPro software (version 6.3; Molecular Devices).

Serum standard, negative, and two replicates of positive controls were included in every test plate. Nunc Maxisorp (Nunc^®^), Grand Island, NE, USA; flat-bottom microtiter plates were coated with 2.7 µg/mL of HPV16 L1 VLP in PBS, covered, and stored at 4 °C until use. On the day of the assay, the plates were washed in Washing Buffer (PBS (Gibco/Life Technologies, Grand Island, NE, USA), pH 7.0 with 1.7 M NaCl (EMD Millipore, Burlington, NJ, USA), 30 mM Na_2_HPO_4_ (EMD Millipore), 7 mM KHPO_4_ (Sigma Aldrich, St. Louis, CA, USA), and 0.25% Tween 20 (Sigma Aldrich), then blocked with Blocking Buffer (PBS with 4% dry milk (Difco, Grand Island, NE, USA) and 0.2% Tween 20) for 1.5 h. After washing, samples were diluted by performing 2-fold serial dilutions in Blocking Buffer and plated, followed by 1 h incubation at room temperature on a shaker. Starting dilution factors for all samples and negative control were 1:100. The plates were washed and one of the following HRP-conjugated secondary antibodies was plated with the samples: goat anti-human total IgG antibody (KPL, Milford, CO, USA; cat# 214-1002); mouse anti-human IgG1 (Southern Biotech, Birmingham, AL, USA; cat# 9052-05), mouse anti-human IgG2 (Southern Biotech; cat# 9070-05), mouse anti-human IgG3 (Southern Biotech; cat# 9210-05), mouse anti-human IgG4 (Southern Biotech; cat# 9200-05), goat anti-human IgA (Jackson ImmunoResearch, West Grove, PA, USA; cat# 109-035-011), or goat anti-human IgM (Jackson ImmunoResearch, West Grove, USA; cat# 109-035-129). Following 1 h incubation on a shaker at room temperature, plates were washed and then substrate (3, 5, 3′, 5′-tetramethylbenzidine (TMB)) (KPL, Milford, CO, USA; cat# 50-73-03) added. Reaction was developed for 25 min at room temperature in the dark, followed by the addition of 0.36 N H_2_SO_4_ (J. T. Baker, Phillipsburg, NJ, USA; cat# 4700-01) to stop the reaction. OD was measured at 450 and 620 nm with a plate reader (SpectraMax i3, Molecular Devices, San Jose, CA, USA) using SoftMax Pro software (version 6.3).

### 2.5. Assessment of Assay Reproducibility and Measurement of Antibody Isotype Levels in CVT Samples

Ninety-five CVT samples from 2vHPV-vaccinated participants were categorized into low-, medium-, and high-concentration groups based on the measured total IgG concentrations (EU/mL). To assess assay reproducibility, 72 of these 95 samples were utilized: 24, 25, and 23 samples from the low-, medium-, and high-concentration groups, respectively. Each sample was tested in duplicate on the same plate, tested on 2 separate days on plates that were prepared on 2 different days, and tested by 2 different technicians on the same day, resulting in 8 replicate tests per sample.

The remaining 23 of the 95 samples were measured (1 measurement) for each antibody type after the reproducibility tests were completed. The results from all 95 samples for each assay were combined for analysis using the mean of the 8 replicates for the 72 samples from the reproducibility tests.

### 2.6. Statistical Analysis

The replicate results from the reproducibility tests were used to determine CVs and ICC using SAS (SAS PRO GLM). Analyses were conducted overall and separately for the low-, medium-, and high-concentration groups. CVs were calculated overall and specifically for between-technician and across-day variability for the 3 concentration groups. Additional descriptive and inferential statistics, including Pearson correlation coefficients, *t*-test (unpaired, 2-tailed), and Fisher’s exact tests, were calculated using GraphPad Prism (version 7) or SAS version 9.3.

## 3. Results

### 3.1. Optimization of Serum Standards

IgG1, IgG3, IgA, and IgM were detected in serum standard, as well as in the OHS serum samples collected from self-reporting vaccine recipients. IgG2 and IgG4 were not detected at appreciable levels in serum but were detectable in purified total IgG that was isolated from an OHS sample known to contain high levels of anti-HPV16 L1 total IgG antibodies (Table S1). When serum standard was tested, there was very little to no detection of IgG2 or IgG4 (Table S1). These findings indicated that the anti-IgG2/IgG4 secondary antibodies were functional, but levels of antigen-specific IgG2 and IgG4 antibodies were too low in serum for detection. Hence, these subclasses of IgG were not pursued further in this study.

### 3.2. Concentrations of HPV16 L1-Specific Total IgG

To assess assay reproducibility, serum samples were selected from the CVT study representing a wide range of total IgG concentrations that could be divided into low-, medium-, and high-concentration groups. Participants were adult women, ages 18–25 years, who had received one dose of 2vHPV (collected 1 month post-vaccination; N = 70) or three doses (collected 12 months post-first vaccination; N = 25) of 2vHPV.

HPV16 L1-specific total IgG was measured in these 95 samples and in the 25 pre-vaccinated samples that were collected from participants who at the enrollment were HPV16/18 seronegative and negative for HPV DNAs. As expected, the naive samples did not have detectable levels of total IgGs. However, a broad range of antibody concentrations were detected in the 95 samples collected from the post-vaccination time points (range: 22 to 14,656 EU/mL; Table S2). Samples were categorized into low-, medium-, and high-concentration groups (Table S2) for subsequent analyses of the data from reproducibility tests. The first 70 samples were dichotomized based on the median measured concentration (239.5 EU/mL). These samples were classified into low (<240 EU/mL; N = 35) or medium (240–1000 EU/mL; N = 35) concentration groups. The former group had a median concentration of 139 EU/mL (range: 22–237), and the latter group had a median of 389 EU/mL (range: 242–978) (Table S2). The last 25 samples were categorized into the high-concentration group, which had a median of 1757 (range: 1093–14,656) (Table S2).

### 3.3. Evaluation of Assay Reproducibility for IgG1, IgG3, IgA, and IgM ELISAs

**Figure 1:**
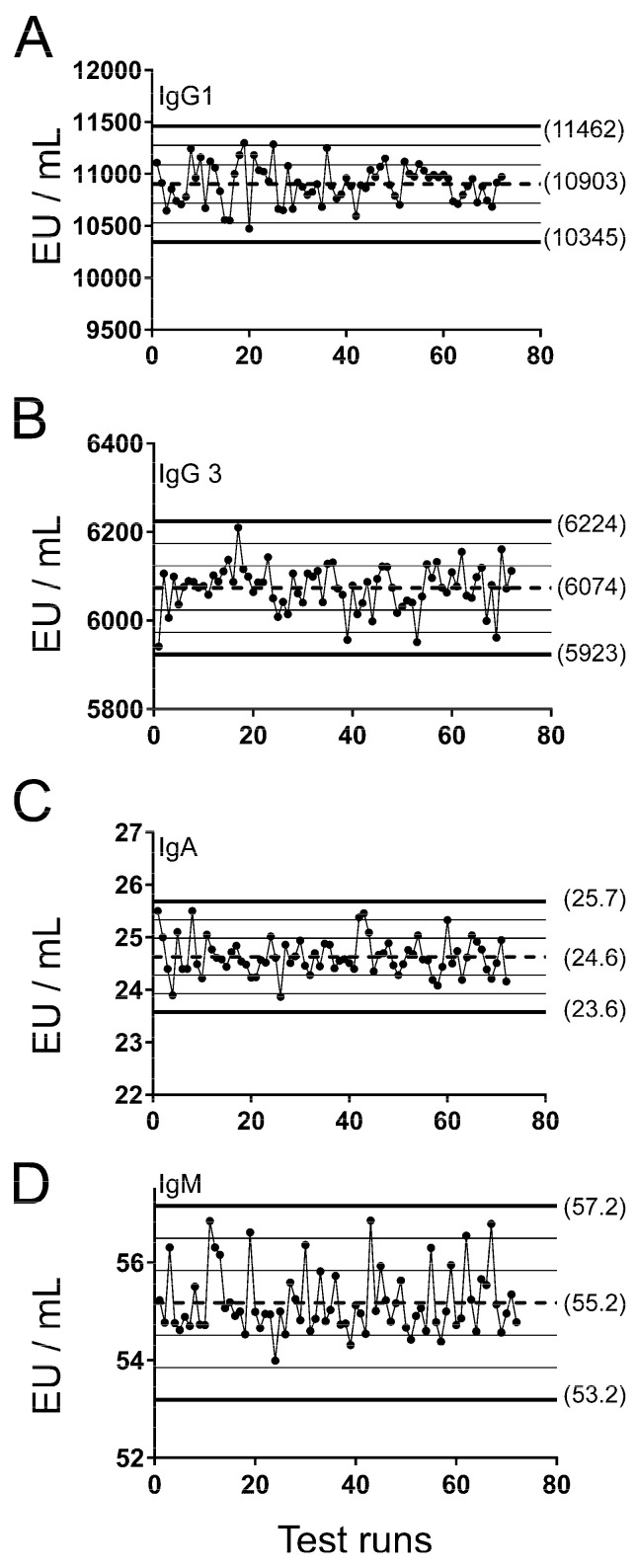
Levey–Jennings plots for serum standards used in IgG1, IgG3, IgA, and IgM ELISAs. Calculated concentrations of serum standards in EU/mL from plates during reproducibility tests were plotted for (**A**) IgG1, (**B**) IgG3, (**C**) IgA, and (**D**) IgM. Mean (dotted line), 1 ± SD (thin solid lines), 2 ± SD (thin solid lines), and 3 ± SD (thick solid lines) are shown in each plot. Standard was tested a combined 72 times over several days by two technicians.

**Figure 2:**
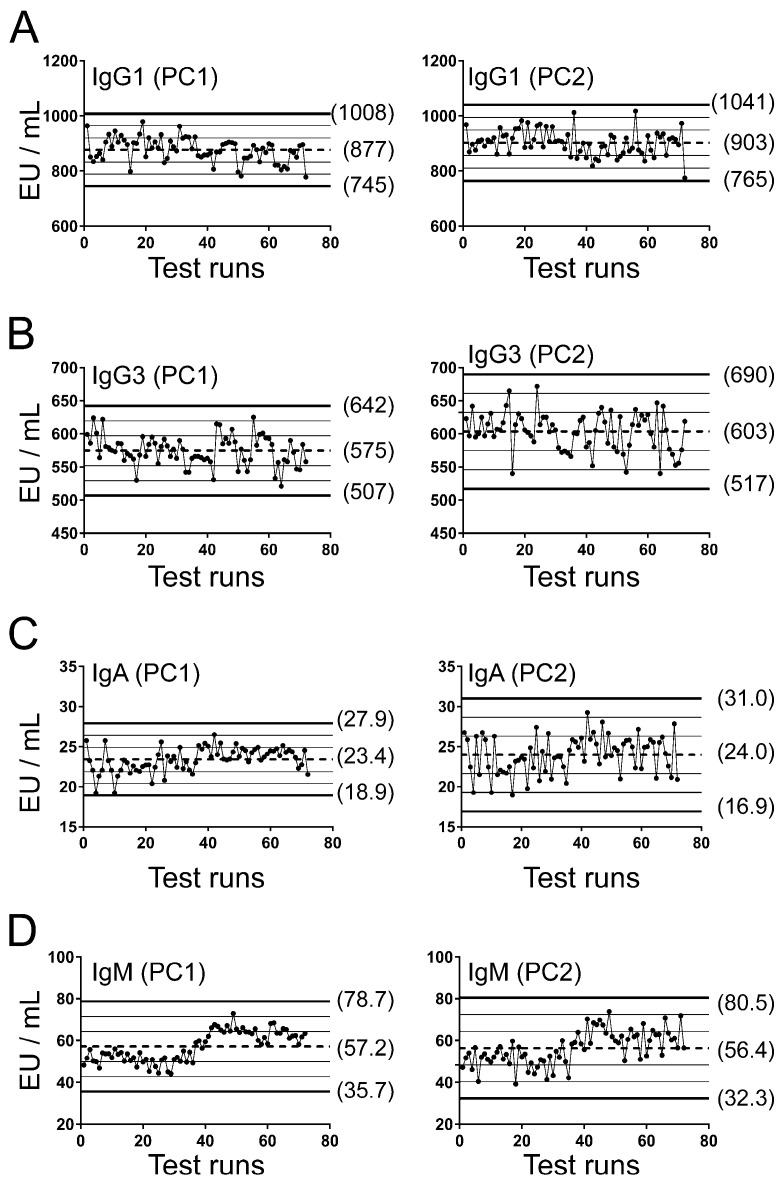
Levey–Jennings plots for positive controls used in IgG1, IgG3, IgA, and IgM ELISAs. On each plate, the same positive control was tested in different columns (PC1 and PC2). The concentrations of the controls were plotted for (**A**) IgG1, (**B**) IgG3, (**C**) IgA, and (**D**) IgM. Mean (dotted line), 1 ± SD (thin solid lines), 2 ± SD (thin solid lines), and 3 ± SD (thick solid lines) are shown in each plot. Each control (PC1 and PC2) was tested a combined 72 times over several days between two technicians.

After measuring and categorizing anti-HPV16 L1-specific total IgG levels in the CVT samples, reproducibility assessments were performed for IgG1, IgG3, IgA, and IgM ELISAs, starting with serum standards (Figure 1) and positive controls (Figure 2) measured by two different technicians using Levey–Jennings plots. The two positive controls (PC1 and PC2) were separately plotted. In both figures, the horizontal lines indicate the mean (dotted lines) and ±1, 2, and 3 SD (solid lines) of the mean. Overall, the performances of standards and controls were acceptable, all values were within ±3 SD, and most within ±2 SD (≥91.7% for all; range: 91.7–98.6%). The controls for IgM, and to some extent for IgA, did drift slightly over time, but not to a level that affected performance.

Further testing of the reproducibility of the ELISAs was performed using 72 of the 95 post-vaccination samples. The concentrations of different antibodies in the 72 test samples are listed in Table 1 as means of the eight replicates. The results are summarized in Table 2. For all assays, the overall ICC values were high (≥98.7%), and they remained high (≥98.5%) when evaluated separately based on the low-, medium-, and high-concentration groups. The overall CV values were 10% or less (range: 7.7–10.0%) for all the assays except for IgM ELISA, which had an overall CV of 31.1% (Table 2). This was partially due to one outlier (#35, Table 1) with a much higher concentration (173.4 EU/mL) than the remaining samples. When this sample was excluded, the overall CV estimate dropped to 17.4%. When CVs were evaluated separately based on the total IgG concentration groups (low, medium, and high), variations were also low for ELISAs for IgG1 (range: 5.0–6.3%), IgG3 (range: 5.6–8.4%), and IgA (range: 6.6–10.5%) (Table 2). For the IgM assay, the CVs ranged from 9.5% to 27.6%. Between-technician CVs were low to moderate for all categories tested for all assays, and across-day CVs were consistent with the overall findings (Table 2).

### 3.4. Pattern of Antibody Responses among Vaccinated Individuals

To obtain a complete profile of different antibodies in all 95 CVT samples, the remaining 23 samples were analyzed for IgG1, IgG3, IgA, and IgM and stratified by the low, medium, and high total IgG concentration groups. Overall detectability was 86.3% (IgG1), 100% (IgG3), 88.4% (IgA), and 62.1% (IgM) (Table 3). There were 13 IgG1, 11 IgA, and 36 IgM samples that were below the detection level. For those with detectable levels of antibodies, the concentration ranges were 31–13,344 EU/mL for IgG1 (median = 498); 10–6055 EU/mL for IgG3 (median = 156); 0.55–20.46 EU/mL for IgA (median = 1.97); and 4.85–173.4 EU/mL for IgM (median = 10.77). Antibody concentrations plotted as a function of time demonstrated the concentrations were higher in 12 months post-first dose samples than those in 1 month post-first dose samples for all antibodies with the exception of IgM, which was lower (Figure S1).

### 3.5. Correlation between Individual Antibody Types and Total IgG

**Figure 3:**
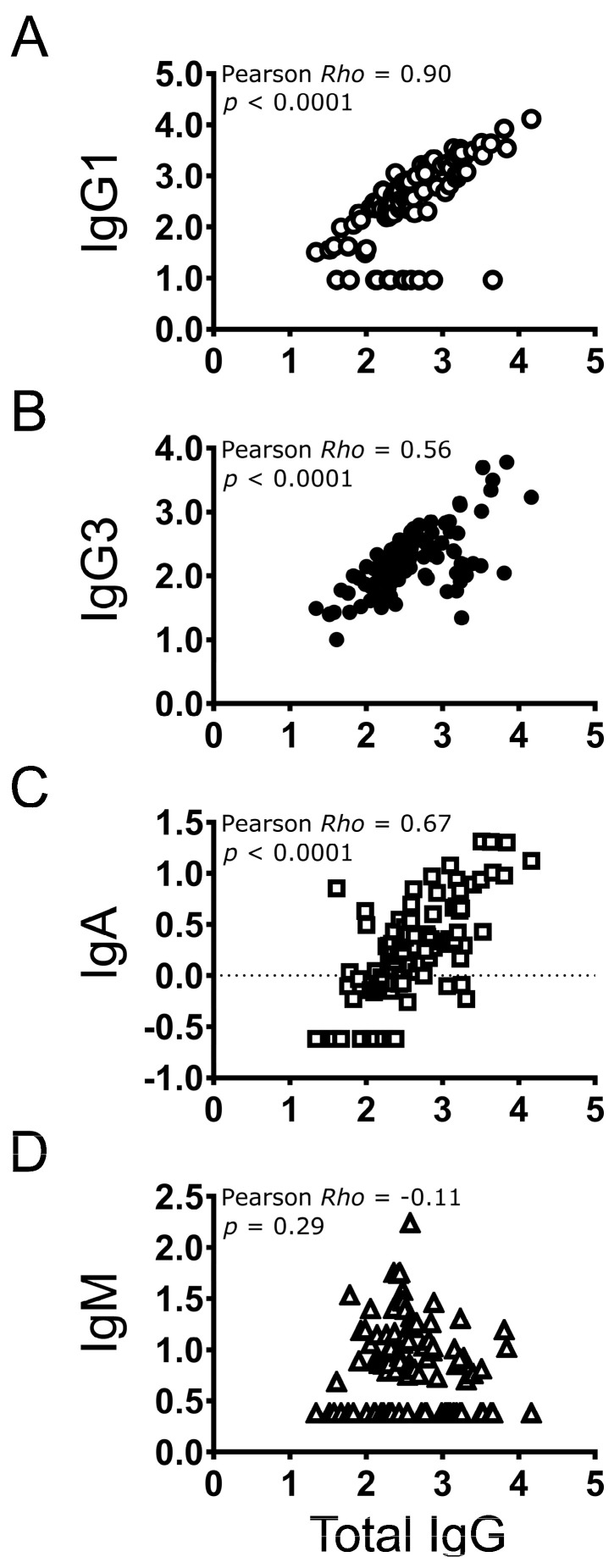
Correlation analyses of antibody concentrations between total IgG vs. IgG1, IgG3, IgA, and IgM. Measured antibody concentrations in EU/mL units were log transformed (log10) and plotted. Pearson *r* and *p* values are shown (N = 95). Measured antibody concentrations in EU/mL units were log transformed (log10) and plotted. Pearson *r* and p values are shown (N = 95).  Pearson correlational analyses between total IgG and the respective isotype antibody level (IgG, IgG3, IgA, or IgM) were plotted (**A**–**D**).  The correlations were statistically significant between total IgG and IgG1, IgG3, or IgA, but were not significant between total IgG and IgM.

IgG1, IgG3, and IgA antibody levels increased as total IgG concentrations increased (low, medium, and high; Table 3), but the opposite was observed for IgM (Table 3). The strongest correlation was observed between total IgG and IgG1 (Pearson *r* = 0.90, *p* < 0.0001) (Figure 3A). Significant but more modest levels of correlation were observed between total IgG and IgG3 (Pearson *r* = 0.56, *p* < 0.0001; Figure 3B) or IgA (Pearson *r* = 0.67, *p* < 0.0001; Figure 3C). A low, negative correlation was observed between total IgG and IgM (Pearson *r* = −0.11, *p* = 0.29; Figure 3D).

A broad range of total IgG concentration was observed in IgG1-negative individuals (N = 13; range: 41–4558 EU/mL). Twelve of the thirteen samples were from 1 month post-first dose collection time point, and one was from 12 months post-first dose. These 13 IgG1-negative individuals did not differ significantly in total IgG levels compared to IgG1-positive individuals (Mann–Whitney test, *p* = 0.18). Age, BMI, or oral contraceptive usage showed no associations in these two groups. However, the percentage of smokers (current or past) was higher in the IgG1-negative group (Fisher’s *p* value = 0.04). The correlation between total IgG and IgG3 was 0.99 among the 13 IgG1-negative individuals (*p* < 0.0001, Table S3).

The 11 individuals with no detectable levels of IgA had lower total IgG levels (median 115 EU/mL) than the 84 individuals with detectable IgA (median 389 EU/mL) (Mann–Whitney test, *p* < 0.0001). There were no differences between these two groups with respect to age, BMI, smoking, or oral contraceptive usage. All 11 IgA-negative individuals were from the 1 month post-first dose collection time point and had only received one dose of vaccine; ten were in the low total IgG concentration group, and one was in the medium-concentration group.

### 3.6. Correlation amongst Antibody Isotypes in Vaccinated Individuals

**Figure 4:**
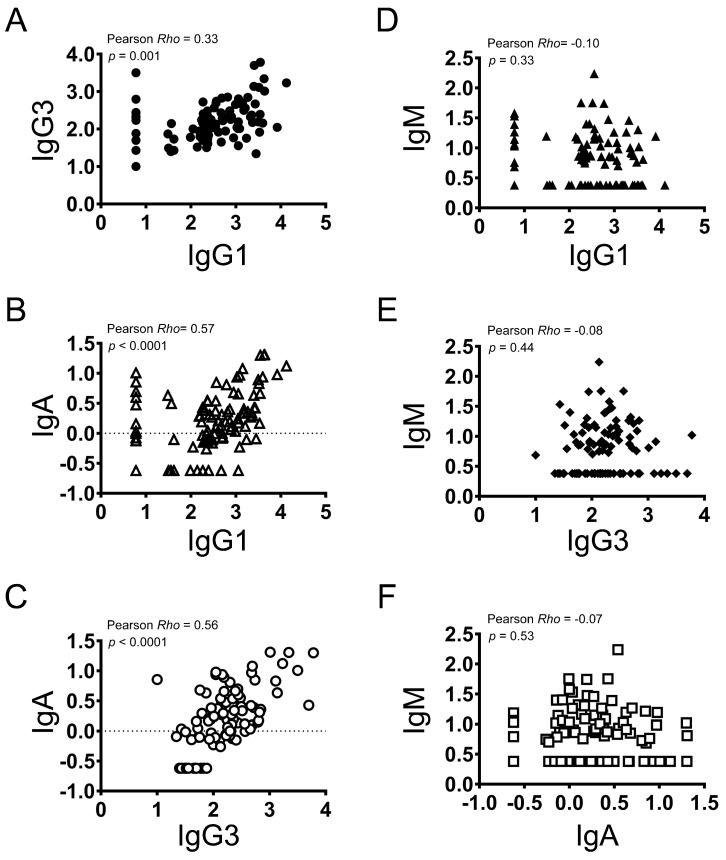
Correlation analyses of antibody concentrations between different antibody types. Measured antibody concentrations in EU/mL units were log transformed (log10) and plotted. Pearson *r* and *p* values are shown (N = 95). Pearson correlational analyses between IgG1 and IgG3, IgA, or IgM are plotted (**A**–**F**).  Also, correlational analyses between IgG3 and IgA or IgM and between IgA and IgM are plotted. The correlations were statistically significant between IgG1 and IgG3 or IgA, and between IgG3 and IgA.  Between IgG1 and IgM, IgG3 and IgM, and IgA and IgM were not significantly different.

Significant correlations (range: 0.33–0.57; *p* ≤ 0.001 for all) were observed between IgG1, IgG3, and IgA concentrations (Figure 4A–C), but these correlations tended to be lower than those observed between these antibodies and total IgG. A modest level of correlation was observed between IgG1 and IgG3 (Pearson *r* = 0.33, *p* = 0.001; Figure 4A). Exclusion of the 13 individuals who tested negative for IgG1 did not materially affect the level of correlation observed between IgG1 and IgG3 (Pearson *r* = 0.36, *p* = 0.0008; Table S4).

No correlations were observed between IgM and IgG1, IgG3, or IgA (range in Pearson *r*: −0.10 to −0.07; *p* > 0.33 for all; Figure 4D–F).

## 4. Discussion

Secreted IgM antibodies are among the first isotypes found in the serum as part of the humoral response to a foreign antigen, followed by higher-affinity isotypes IgG and IgA [27,28]. In humans, there are four different subclasses of IgGs (IgG1, IgG2, IgG3, and IgG4) and two subclasses for IgA [29,30]. The interactions between antibodies and Fc receptors are known to influence protection afforded through humoral- and cell-mediated immunity [31,32,33]. Secreted IgM antibodies, which are typically found in the blood or mucosa, conform into large pentameric IgM molecules, resulting in high avidity (overall binding strength to an antigen) against pathogens with repetitive epitopes [28]. Examples of targets with repetitive epitopes can include bacterial capsules or virus-like particles (VLPs) such as those used in the HPV vaccines [28,34]. Meanwhile, IgG isotypes IgG1 and IgG3 are known to have particular anti-viral functions [35,36], and IgA plays a crucial role in mucosal immunity [29,37,38]. Consequently, examination of different antibodies induced by HPV vaccination may provide useful insights into why these vaccines are so highly efficacious, even at a reduced number of doses.

Although there are some published studies that reported on HPV-specific antibody isotypes [17,39,40,41,42,43,44,45], this is an area of research that remains relatively unexplored, particularly in the context of extended follow-up time and number of vaccine doses received. Some of the reported studies have examined the presence of antibody isotypes in the context of HPV infections [44], and in HPV-infected individuals who were either HIV positive or negative [40]. Antibody isotype profiles were measured in rhesus macaques that were immunized with VLPs of different HPV types, including HPV16 [41]. Isotypes were also previously measured in a small trial in humans vaccinated with an in-house HPV16 L1 VLP [39] and later studied in participants either naturally infected or vaccinated with 2vHPV [42]. The first demonstrated high levels of IgG1, low levels of IgG2, and weak or variable IgG3 and IgG4 responses to vaccination [39]. Scherpenisse et al., however, showed IgG1 and IgG3 to be the most abundant subtypes in both the naturally infected and the vaccinated cohorts [42]. More recently, antibody isotypes were quantified in girls that had received one, two, or three doses of the 2vHPV vaccine, revealing that all three dosing schedules produced similar isotype proportions, with the highest being IgG1, followed by IgG3 and very low amounts of IgG2 and IgG4 [17].

In this study, we developed ELISAs to measure concentrations of IgG1, IgG3, IgA, and IgM antibodies against HPV16 L1. Using sera from the CVT study that covered a broad range of total IgG concentrations, we demonstrated generally high reproducibility for all assays. ICCs were invariably high and CVs low with the exception of the IgM assay, for which the overall CVs were on the order of 30%. However, this was partially due to one outlier sample. Although the observed variability across technicians was higher than that observed across time for a given technician, it was still generally low to moderate. Overall, all assays performed well and were reliable when performed by different technicians on different days. A previous study was published detailing an ELISA capable of distinguishing between IgG and IgM that was used to measure antibody isotype levels in girls (15–19 years of age) previously vaccinated with zero, one, two, or three doses of the 4vHPV vaccine [43]. However, antibody subtypes (IgG1, IgG2, IgG3, and IgG4) and IgA were not measured, and the assay was not validated using a large independent cohort as is reported in this paper [43]. In the future, we are considering integrating automation as well as multiplex platforms in order to increase ELISA output and facilitate increased testing of HPV antibody isotypes.

Our data on the detection of subclasses of IgGs are in agreement with previous studies in which IgG1 and IgG3 were the major subclasses of IgG detected in 2vHPV-vaccinated individuals, and IgG2 and IgG4 were not detected or were very low [17,42]. We were only able to detect IgG2 and IgG4 antibodies after enriching total IgGs using HPV16 L1 VLP coated beads, indicating that these subclasses of IgG can be generated in response to 2vHPV but at low levels such that it is difficult to detect these antibodies in serum. It was not possible for us to test IgG2 and IgG4 in the CVT serum test samples using HPV16 L1 VLP coated beads, primarily because the volume of the samples was limited. In addition, low levels of detectability for IgG2 and IgG4 in serum samples are consistent with the notion that IgG2 is predominantly induced in response to polysaccharides, such as in bacteria [30,46,47], and that IgG4 is typically observed in response to repeated exposure to an antigen, such as allergens [30]. Our data also showed that total IgG, IgG1, IgG3, and IgA, but not IgM, antibody levels were higher in the 12-month samples than those in the 1-month samples. Such results would be expected as the IgM level is generally at its highest during the first few days after exposure to an antigen and declines thereafter, while IgG and IgA levels are known to increase as the immune response matures [27,28]. These results further indicate that these ELISAs perform well and can be used to measure serum IgG1, IgG3, IgA, and IgM.

Correlational analyses suggested that, in general, higher levels of total IgG occurred alongside higher levels of IgG1, IgG3, and IgA, but not with IgM. These results may be suggesting that measuring total IgG could be sufficient for monitoring of antibodies elicited in response to 2vHPV. However, there are few data available on the relationship between total IgG and IgG subclasses, or IgA, as a function of time and the number of doses received. Studies of the kinetics of HPV isotypes after infection and different doses of vaccines are of interest in the future. Therefore, further testing to accumulate more data will be needed.

The observation that 13 vaccine recipients from the CVT samples did not mount IgG1 responses but were IgG3 positive was of interest. As 12 of 13 IgG1-negative samples were from the 1 month post-first dose collection time point, it remains to be determined whether a longer lapse in time after the initial immunization will lead to generation of IgG1 antibodies before receiving additional doses of vaccines. The percentage of smokers (current or past) was higher in the IgG1-negative group, however, it is currently unknown how smoking could affect the IgG1 level. As the sample size was only 13, more data will need to be accumulated to better understand whether smoking affects the level of IgG1 in vaccine recipients. Only one IgG1-negative sample was from 12 months post-first dose in an individual who had received all three doses of 2vHPV. Currently, it is not clear why this individual failed to generate detectable level of IgG1 even after receiving three immunizations.

It is worth noting that all 11 IgA-negative samples were from the 1 month post-first dose collection time point and had received only one dose of 2vHPV. This finding is in agreement with Pasman et al., who reported that the IgA response was significantly lower in the one-dose cohort at the 5-year time point compared to the two- and three-dose cohorts [17].

In addition to monitoring isotype levels in the context of vaccine studies, it would be also interesting to evaluate the relationship between detection and levels of anti-HPV isotypes and protection against HPV infection and cervical cancer development in natural history studies of HPV infection. These studies may contribute to a better understanding of humoral immune responses developed in the context of infection and vaccination.

## 5. Conclusions

Moving forward, these assays could be used to explore important questions pertaining to how the number of doses of HPV vaccines influences the generation and persistence of specific antibody isotypes. As the number of doses of the vaccines administered might be critical to generate a complete set of long-lasting, protective antibodies, it will be important to test samples from longitudinal studies to examine how the profiles of these antibodies might change over time. As immunization with HPV vaccines can generate cross-neutralization effect against HPV types that are phylogenetically related to HPV16 and HPV18 [15,48], it will also be of interest to investigate whether cross-neutralization is a generalized phenomenon or mediated through specific antibody isotypes.

## Figures and Tables

**Table 1:** The mean concentrations (EU/mL) of IgG1, IgG3, IgA, and IgM in the seventy-two CVT samples. Mean concentrations of different antibodies in the seventy-two CVT samples are listed. The means of eight replicates are shown. Twenty-four, twenty-five, and twenty-three samples from the low-, medium-, and high-concentration groups, respectively, were tested. The grouping was based on the concentration of anti-HPV16 L1-specific total IgG (Table S2). The italicized numbers in parentheses are those samples with non-detectable levels of the antibodies. For these samples, half the cut-off values were assigned: IgG1 (6 EU/mL); IgG3 (0.625 EU/mL); IgA (0.24 EU/mL); IgM (2.40 EU/mL). IgG3 was detected in all samples.

N	IgG3	IgG1	IgA	IgM
1	31	32	(*0.24*)	(*2.40*)
2	25	36	(*0.24*)	(*2.40*)
3	27	42	(*0.24*)	(*2.40*)
4	10	98	(*0.24*)	(*2.40*)
5	60	42	0.79	(*2.40*)
6	54	112	0.59	(*2.40*)
7	27	182	0.93	7.74
8	100	139	(*0.24*)	15.31
9	41	238	0.72	25.07
10	68	247	(*0.24*)	11.49
11	129	299	0.69	(*2.40*)
12	76	236	1.11	8.01
13	53	303	0.78	7.30
14	217	224	0.97	(*2.40*)
15	108	516	0.87	7.16
16	32	474	(*0.24*)	(*2.40*)
17	50	160	1.96	7.26
18	63	174	1.56	(*2.40*)
19	175	184	(*0.24*)	(*2.40*)
20	48	189	0.71	10.42
21	50	262	2.06	(*2.40*)
22	255	409	1.62	(*2.40*)
23	114	281	0.84	25.36
24	83	183	2.69	56.53
25	88	591	1.56	55.43
26	194	499	0.83	(*2.40*)
27	302	233	3.54	8.56
28	367	329	0.99	56.76
29	139	294	1.87	7.65
30	205	737	1.58	29.75
31	170	800	1.29	(*2.40*)
32	227	222	0.55	5.61
33	305	735	2.52	12.28
34	180	773	1.16	7.26
35	135	359	3.48	173.40
36	492	364	1.26	20.91
37	220	237	3.43	6.80
38	272	370	7.03	16.35
39	550	187	2.45	6.39
40	526	985	1.08	18.30
41	198	497	1.00	10.75
42	316	1511	2.54	(*2.40*)
43	92	205	2.41	8.28
44	638	1146	1.49	12.34
45	698	1850	2.29	18.39
46	479	1227	9.34	9.66
47	277	2126	1.87	28.74
48	210	1524	2.17	(*2.40*)
49	198	620	6.44	5.34
50	667	488	2.05	(*2.40*)
51	57	1733	0.79	(*2.40*)
52	711	659	2.29	(*2.40*)
53	496	1421	11.96	(*2.40*)
54	247	3464	4.63	(*2.40*)
55	236	2261	2.19	10.10
56	58	896	4.78	7.10
57	111	1061	8.69	(*2.40*)
58	467	2696	2.64	7.43
59	1376	2526	4.31	8.15
60	1290	3278	6.77	(*2.40*)
61	156	1143	4.54	(*2.40*)
62	22	2821	0.81	(*2.40*)
63	103	1217	0.59	5.04
64	155	3153	7.82	5.79
65	144	3956	8.69	(*2.40*)
66	1026	4355	20.46	6.46
67	5004	2564	2.68	(*2.40*)
68	2202	4260	20.32	(*2.40*)
69	3167	(*6.00*)	10.19	(*2.40*)
70	111	8335	9.53	15.60
71	6055	3511	20.03	10.47
72	1710	13,344	13.26	(*2.40*)

**Table 2:** Intraclass correlation coefficient (ICC) and coefficient of variation (CV) for each ELISA, overall and by the concentration groups. ICCs quantify the proportion of total assay variability due to real, inter-participant differences rather than assay noise. CVs approximated the assay distribution (i.e., mean-to-standard-deviation ratio) and were calculated to analyze the performance of each ELISA system. CVs were calculated overall as well as specific to variation between technicians and variation across testing days. ICCs and CVs were calculated specific to each ELISA using 8 replicates for each study participant (N = 72). Low, medium, and high groups were based on total IgG concentrations (Table S2).

	ICC	Overall CV	Between-Technician CV	Across-Day CV
IgG1				
Overall	99.8	9.0	12.8	6.3
Low	99.7	5.0	5.6	4.0
Medium	99.6	6.3	8.2	4.4
High	99.8	6.1	8.0	4.1
IgG3				
Overall	99.9	7.7	22.7	6.2
Low	99.4	8.4	7.3	7.6
Medium	99.3	6.6	6.8	4.4
High	99.9	5.6	16.2	4.5
IgA				
Overall	99.7	10.0	21.8	9.2
Low	99.0	10.5	7.3	8.0
Medium	99.5	7.6	6.3	6.3
High	99.7	6.6	18.5	6.1
IgM				
Overall	98.7	31.1	15.8	30.6
Low	99.3	15.9	5.6	14.6
Medium	98.5	27.6	14.7	27.3
High	99.2	9.5	12.8	8.3

**Table 3:** Summary of antibody measurements for IgG1, IgG3, IgA, and IgM in the ninety-five CVT samples. Samples with non-detectable levels of antibodies were excluded from the Range, Mean, and Median columns. For the % Detected column, all ninety-five samples were included in the calculations. The low, medium, and high groupings were based on the total IgG levels (Table S2).

		Range (EU/mL)	Mean (EU/mL)	Median (EU/mL)	% Detected
IgG1	All samples	31–13344	1262	498	86.3% (82/95)
	Low	31–516	206	184	82.9% (29/35)
	Medium	187–2126	781	620	82.9% (29/35)
	High	488–13,344	3120	2630	96.0% (24/25)
IgG3	All samples	10–6055	411	156	100% (95/95)
	Low	10–270	92	74	100% (35/35)
	Medium	36–698	286	220	100% (35/35)
	High	22–6055	1032	247	100% (25/25)
IgA	All samples	0.55–20.46	3.56	1.97	88.4% (84/95)
	Low	0.59–7.16	1.63	1.08	71.4% (25/35)
	Medium	0.55–9.34	2.49	1.87	97.1% (34/35)
	High	0.59–20.46	6.94	4.63	100% (25/25)
IgM	All samples	4.85–173.40	17.76	10.77	62.1% (59/95)
	Low	4.85–56.53	15.56	11.49	54.3% (19/35)
	Medium	5.34–173.40	22.34	12.28	82.9% (29/35)
	High	5.04–20.05	9.50	7.79	44.0% (11/25)

## Data Availability

Research data are available upon request to the corresponding author. A trial summary, current publications, and contact information for CVT data access are available online: https://dceg.cancer.gov/research/who-we-study/cohorts/costa-rica-vaccine-trial (accessed on 8 August 2024).

## Supplementary Materials

The following supporting information can be downloaded at: https://www.mdpi.com/article/10.3390/vaccines12101108/s1, Figure S1: Concentrations of different antibody isotypes at 1 and 12 months following the start of the HPV vaccination series; Table S1: Detectability of IgG2 and IgG4; Table S2: Measured CVT sample concentrations of anti-HPV16 L1-specific total IgG; Table S3: Correlational analyses between total IgG, IgG3, IgA, and IgM in the thirteen IgG1-negative and eighty-two IgG1-positive samples; Table S4: Pearson correlational analyses between IgG1 and other antibodies after excluding IgG1-negative samples.
