# Supplementary material for: Reproducibility Assessment of Enzyme-Linked Immunosorbent Assays to Detect Anti-HPV16 L1-Specific IgG1, IgG3, IgA, and IgM Antibodies
Source: Vaccines (Basel). 2024 Sep 27;12(10):1108. doi: 10.3390/vaccines12101108 (PMC11511443; doi:10.3390/vaccines12101108)
Supplement: Supplementary file 1 [file vaccines-12-01108-s001.zip › vaccines-3176180-supplementary.pdf]

Supplemental Materials and Methods

Isolation of total IgG

Approximately 200 mL of blood was collected using BD Vacutainer (BD, cat#:366430) from an OHS donor, and the blood was allowed to clot on ice for 30 minutes. The blood was centrifuged at 1000 × g for 20 minutes at 4°C with the brake off to collect serum. Approximately 90 mL of serum was collected. Eight milliliters of serum were used to isolate total IgG using Protein G agarose beads column (Thermo Fisher Scientific, cat#22852). Slurry of Protein G beads was packed and washed with excess DPBS (Life Technologies, cat#:1897039) in a column.

Eight mL of serum was diluted 4-fold in DPBS, and ran through the Protein G column several times, then, washed in excess DPBS. After washing, total IgG was eluted using 0.5 to 1.0 mL of Pierce IgG Elution Buffer, pH2.0 (Thermo Fisher Scientific, cat# 21028) and collected into tubes containing 100 µL 1M Tris, pH10. Appropriate fractions were pooled and dialyzed against 1 liter of PBS using Slide-A-Lyzer Cassette (MW 20,000 CO; Thermo Fisher Scientific, cat#: 66012). DPBS was exchanged three times over 3 days.

Conjugation of HPV16 L1 VLP, and isolation of HPV16 L1 VLP-specific IgG

HPV16 L1 VLP was conjugated to agarose resins. Four milligrams of HPV16 L1 VLP was dialyzed in PBS (0.1M sodium phosphate, 0.15M NaCl, pH7.2; BupH Phosphate Buffered Saline Packs, Thermo Fisher Scientific, cat#: 28372). Three hundred milligrams of Pierce NIH-activated Agarose Resin (Thermo Fisher Scientific, cat#:26196) was used to mix with 4 mL of HPV16 L1 VLP in a Pierce Centrifuge column (Thermo Fisher Scientific, cat#: 89898). The capped column was put on a

rotator for 2 hours at room temperature. Then, the column with the VLP/resin was put inside a 50 mL conical tube and centrifuged for 1 minute at 1000 × g. The VLP/resin in the column was washed twice with 4 mL of PBS by centrifugation at 1000 × g for 1 minute. Then, 4 mL of 1M Tris, pH7.5 was added and the column was put on a rotator for 20 minutes at room temperature. Tris buffer was eluted by centrifugation. The column was washed twice more with the 1M Tris buffer, then, the column was washed with 2 mL of PBS three times.

To enrich the isolated total IgGs, antibodies were mixed with the HPV16 L1 VLP conjugated agarose resin, and incubated overnight on a rotator at 4°C. Next day, the column was washed three times with 6 mL of PBS, and the bound antibodies were eluted with 0.5 - 1 mL of Pierce IgG Elution Buffer, pH2.0 and collected into tubes containing 100 µL1M Tris, pH10. The appropriate fractions were pooled, and dialyzed against 1 liter of PBS, the PBS was exchanged three times over 3 days.

S1 Figure

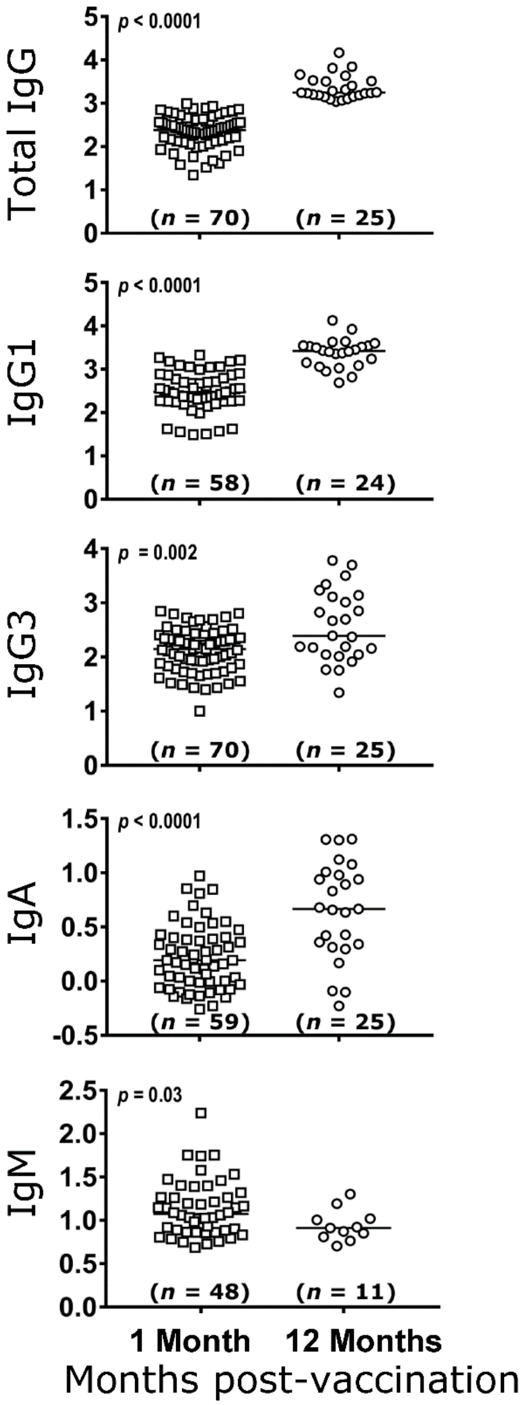

Figure S1. Concentrations of different antibody isotypes at 1- and 12-months following the start of the HPV vaccination series. Concentrations (EU/mL) of antibody isotypes were log-transformed (Log_{10}) and plotted as a function of time. Samples with detectable levels of antibodies were plotted. Unpaired, one-sided Mann-Whitney tests were performed, and *p* values are shown. Bars indicate medians.

Table S1. Detectability of IgG2 and IgG4

	O.D.	
Amount of antigen-column purified IgGs (µg)*	IgG2	IgG4
60	0.8376	2.8902
30	0.4117	1.8799
15	0.2577	1.0302
7.5	0.1611	0.6008
3.5	0.1593	0.2912
1.875	0.0886	0.1541
0.9375	0.0232	0.0943
0.46875	0.0166	0.0484
Dilution factors of serum standard	IgG2	IgG4
100	0.0163	0.2669
200	0.0166	0.3259
400	0.0146	0.2636
800	0.0115	0.1373
1600	0.0068	0.0896
3200	0.0047	0.0246
6400	0.0025	0.0174
12,800	0.0029	0.0096

*Total IgG from an OHS sample was purified with a Protein G column first. Eluate from this column was incubated with beads coated with HPV16 L1 VLPs. Eluate from the beads was used as a sample, and ELISAs were performed to detect IgG2 or IgG4. O.D., optical density.

Serum standard we used for the study was also tested. The starting dilution of the serum was 1:100. Total IgG, IgG1, IgG3, IgA, and IgM were detected in this serum standard using the starting dilution factors of 1:8400, 1:4200, 1:3150, 1:200, and 1:300, respectively.

HRP-conjugated anti-IgG2 or -IgG4 secondary antibody was used at 0.8 µg/mL and 1 µg/mL, respectively.

Table S2. Measured CVT sample concentrations of anti-HPV16 L1 specific total IgG.

N	Group	Total IgG (EU/mL)	Time Point (Month)
1		22	1
2		33	1
3		38	1
4		41	1
5		47	1
6		58	1
7		61	1
8		68	1
9		80	1
10		85	1
11		97	1
12		101	1
13		114	1
14		115	1
15		127	1
16		131	1
17		133	1
18	Low	139	1
19		148	1
20		158	1
21		166	1
22		168	1
23		184	1
24		188	1
25		198	1
26		199	1
27		199	1
28		208	1
29		210	1
30		212	1
31		217	1
32		223	1
33		229	1
34		231	1
35		237	1
36		242	1
37		265	1
38		271	1
39		274	1
40		275	1
41		281	1
42		302	1
43		302	1
44		303	1
45		322	1
46		323	1

47		350	1
48		350	1
49	Medium	360	1
50		368	1
51		378	1
52		384	1
53		389	1
54		389	1
55		418	1
56		430	1
57		457	1
58		492	1
59		534	1
60		567	1
61		594	12
62		619	1
63		634	1
64		666	1
65		705	1
66		728	1
67		751	1
68		774	1
69		847	12
70		978	1
71		1093	12
72		1157	12
73		1230	12
74		1262	12
75		1403	12
76		1434	12
77		1530	12
78		1533	12
79		1586	12
80		1685	12
81		1703	12
82	High	1718	1
83		1757	12
84		1780	12
85		1904	1
86		2057	12
87		2521	12
88		3192	12
89		3247	12
90		3376	12
91		4326	12
92		4558	12
93		6457	12
94		6954	12
95		14,656	12

Ninety-five CVT samples from 2vHPV immunized participants were measured for HPV16 L1-specific total IgG levels by ELISA. The numbers in the Time Point column indicate the month at which the samples were collected after the participants had received the initial dose of 2vHPV. Samples collected after one (N=70) or twelve months (N=25) post-first vaccination were tested. Samples were collected from different individuals; therefore, these were not longitudinal samples. We did not detect HPV16 L1-specific total IgG in the naive (HPV DNA and sero-negatives) samples (N=25). Total IgG was detected in all samples from the vaccinated participants.

Table S3. Correlational analyses between total IgG, IgG3, IgA, and IgM in the thirteen IgG1-negative, and eighty-two IgG1-positive samples.

	Pearson *rho* (N = 13, IgG1 negative)	Pearson *rho* (N = 82, IgG1 positive)
Total IgG vs IgG3	0.99	0.53
Total IgG vs IgA	0.74	0.66
Total IgG vs IgM	-0.29	-0.10
IgG3 vs IgA	0.71	0.55
IgG3 vs IgM	-0.30	-0.06
IgA vs IgM	-0.29	-0.05

Pearson correlational analyses were performed on the thirteen IgG1-negative samples, and on the eighty-two IgG1-positive samples. All correlations were statistically significant with $p \leq 0.006$ for all, except when compared with IgM ($p > 0.33$).

Table S4. Pearson correlational analyses between IgG1 and other antibodies after excluding IgG1 negative samples.

	Pearson rho (*N* = 82)	Pearson rho (*N* = 95)
IgG1 vs total IgG	0.93	0.90
IgG1 vs IgG3	0.36	0.33
IgG1 vs IgA	0.60	0.57
IgG1 vs IgM	-0.10	-0.10

Pearson correlational analyses were performed without the thirteen samples that did not have detectable levels of IgG1. These correlations showed statistical significance for all (vs IgG3, $p = 0.0008$; vs IgA or vs. total IgG, $p < 0.0001$), except for IgM ($p = 0.39$). The Pearson rho values for all 95 samples are also shown for comparison, and the p values for these are shown in Fig. 3 (IgG1 vs total IgG), and Fig.4 (IgG1 vs IgG3, IgA, or IgM).
